# Protection against Influenza A Virus Challenge with M2e-Displaying Filamentous *Escherichia coli* Phages

**DOI:** 10.1371/journal.pone.0126650

**Published:** 2015-05-14

**Authors:** Lei Deng, Lorena Itatí Ibañez, Veronique Van den Bossche, Kenny Roose, Sameh A. Youssef, Alain de Bruin, Walter Fiers, Xavier Saelens

**Affiliations:** 1 Medical Biotechnology Center, VIB, Technologiepark 927, Ghent, Belgium; 2 Department of Biomedical Molecular Biology, Ghent University, Technologiepark 927, Ghent, Belgium; 3 Department of Pathobiology, Faculty of Veterinary Sciences, Utrecht University, Utrecht, The Netherlands; Georgia State University, UNITED STATES

## Abstract

Human influenza viruses are responsible for annual epidemics and occasional pandemics that cause severe illness and mortality in all age groups worldwide. Matrix protein 2 (M2) of influenza A virus is a tetrameric type III membrane protein that functions as a proton-selective channel. The extracellular domain of M2 (M2e) is conserved in human and avian influenza A viruses and is being pursued as a component for a universal influenza A vaccine. To develop a M2e vaccine that is economical and easy to purify, we genetically fused M2e amino acids 2–16 to the N-terminus of pVIII, the major coat protein of filamentous bacteriophage f88. We show that the resulting recombinant f88−M2e2-16 phages are replication competent and display the introduced part of M2e on the phage surface. Immunization of mice with purified f88−M2e2-16 phages in the presence of incomplete Freund’s adjuvant, induced robust M2e-specific serum IgG and protected BALB/c mice against challenge with human and avian influenza A viruses. Thus, replication competent filamentous bacteriophages can be used as efficient and economical carriers to display conserved B cell epitopes of influenza A.

## Introduction

Influenza viruses cause yearly recurrent epidemics and type A influenza viruses can initiate pandemics in humans. Although human influenza can be prevented by vaccination, the economic and clinical burden of human influenza is still high [1, 2]. Licensed seasonal influenza vaccines can prevent or reduce flu symptoms in children, adults and the elderly, although their benefit for the latter group varies [3–6]. These vaccines contain two types of influenza A (H1N1 and H3N2) and one or two serotypes of influenza B. Protection by these vaccines correlates with induction of neutralizing antibodies directed primarily against hemagglutinin of the influenza viruses that are likely to circulate. Vaccine effectiveness varies yearly due to the imperfect anticipation of the nature of the circulating epidemics influenza strain. The composition of seasonal influenza vaccines needs to be reformulated almost every year according to the results of global influenza surveillance networks, coordinated by the World Health Organization [7]. After the WHO makes its recommendations for the next influenza vaccine composition, it takes about six months before the first supplies of approved influenza vaccine becomes available [8]. This delay is particularly worrying if a pandemic outbreak occurs, as most people would be very vulnerable to infection by the pandemic virus, because they lack pre-existing immunity [9]. It is important to mention that vaccine manufacturers have provided proof of concept that this relatively long influenza vaccine production period can be shortened considerably, *e*.*g*. by using synthetic biology and reverse genetics methods to generate the seed viruses for vaccine production [10].

Matrix protein 2 (M2) is the third membrane protein of influenza A viruses. M2 fulfills several important functions in the viral life cycle. It has proton-selective ion channel activity, which is important early after entry of the virion into the host cell [11]. M2 can also activate the inflammasome, subverts autophagy and is involved in virion budding [12–16]. The N-terminal 23 amino acid residues of M2 constitute the ectodomain (M2e). M2e is well conserved among influenza A subtypes, making it an interesting candidate antigen for developing universal influenza A vaccines. But serum antibody responses to M2e are low following experimental infection of animals [17, 18]. In humans, M2-specific serum IgG levels seem to increase with age [19]. Moreover, these M2-specific responses were boosted considerably by natural infection with H1N1 2009 pandemic virus and most clearly so in people who had pre-existing antibodies against M2 [19]. However, whether natural anti-M2e IgG antibodies contribute to protection in human or other influenza A virus hosts remains unclear.

Because of the poor immunogenicity of M2e induced by influenza virus infection, researchers often fused it genetically or chemically with carriers and applied such fusions to induce M2e-specific humoral and cellular responses. Examples of such carriers include virus-like particles [20, 21], cholera toxin A1 subunit [22, 23], keyhole limpet hemocyanin [24], *Lactococcus lactis* [25] and Toll Like Receptor (TLR) 5 agonist flagellin [26, 27], Multiple Antigen Peptide [28], T7 bacteriophage nanoparticles and bacteriophage Qβ [29, 30]. These M2e protein conjugate vaccines were typically combined with adjuvants to induce antibodies and protection against influenza A virus challenge [31].

Protection by M2e-based influenza vaccines is provided mainly by M2e-specific IgG antibodies. Anti-M2e serum passively transferred into naïve laboratory mice provides protective immunity to the recipient animals [20, 31, 32]. M2 is incorporated in low numbers in influenza A virions, yet it is abundantly expressed on the surface of infected cells [33]. The most likely mechanism of action of M2e vaccines is induction of M2e-specific IgGs that bind to M2 on the surface of infected cells, which are subsequently eliminated by antibody-dependent cellular cytotoxicity or by antibody-dependent phagocytosis [34]. Alveolar macrophages and Fcγ receptors are essential for this protection [35]. Notably, M2e-based immunity is infection permissive and does not hamper the induction of cytotoxic T cell responses upon exposure to influenza A virus [18]. This important feature could be advantageous for immunologically naive influenza vaccinees.

In this study, we used the filamentous bacteriophage f88 as a carrier for amino acids 2–16 of M2e. Filamentous bacteriophages are non-lytic viruses that infect and replicate in *Escherichia coli* cells carrying an F episome. Infection with f88 phages slows down bacterial growth but does not kill the host [36]. The minor coat protein pIII, which is involved in host cell recognition and the major coat protein pVIII, which is the most abundant capsid protein, have been frequently used to display heterologous peptides on the phage capsid [37, 38]. We genetically fused M2e2-16 from a human H3N2 virus to the N-terminus of the major coat protein pVIII to generate hybrid phages containing both wild type capsomers and M2e-pVIII capsomers. We show that these phages can be easily purified and can generate M2e-specific systemic IgG responses in mice. Moreover, immunization with these M2e-displaying filamentous phages protected mice against challenge with different influenza A virus subtypes.

## Materials and Methods

Generation, purification and characterization of M2e-displaying f88 bacteriophages

**Fig 1:**
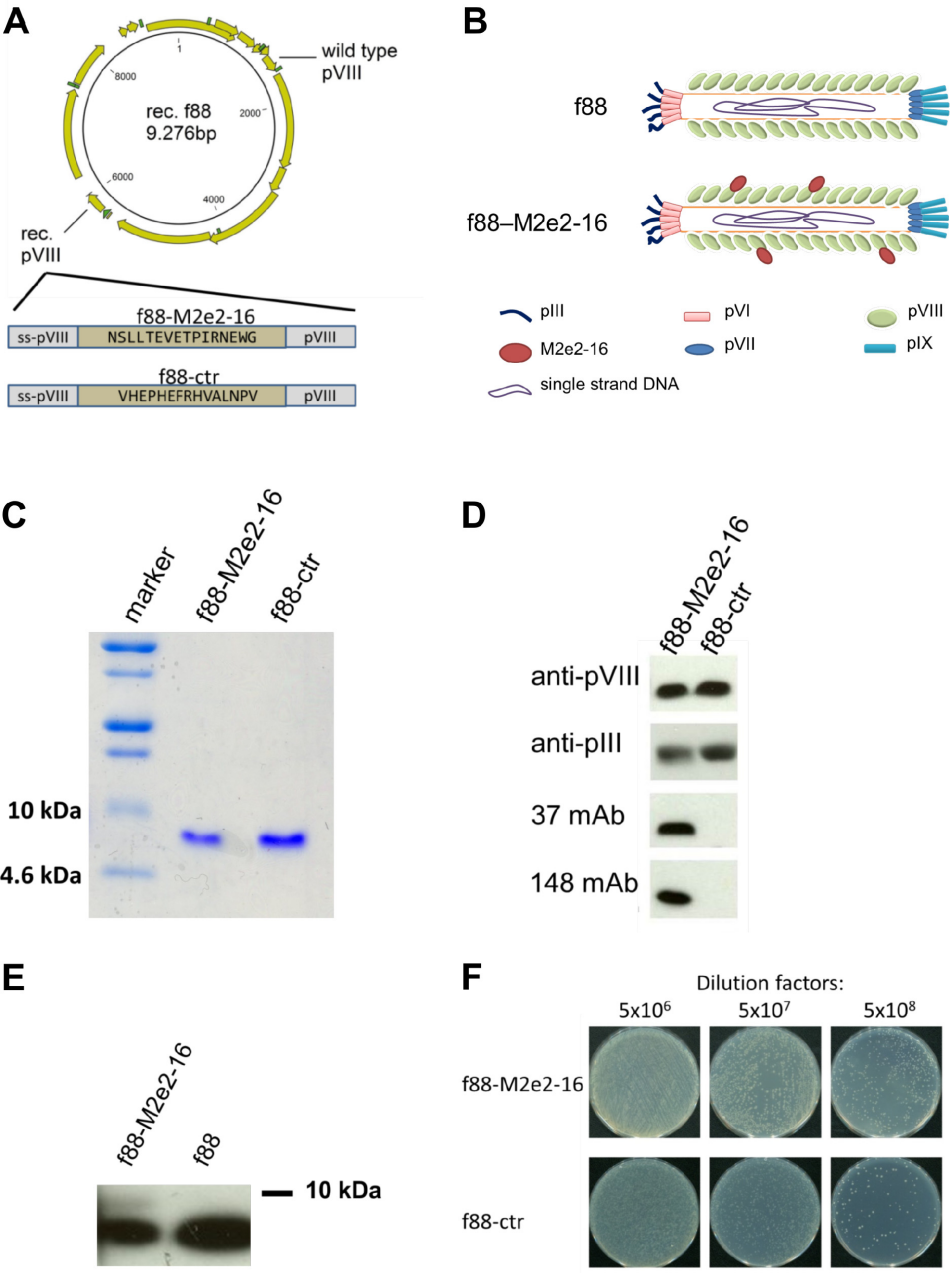
Generation and characterization of M2e-displaying f88 phages. (A) Construct of M2e-displaying or control peptide-displaying f88 plasmid. (B) Schematic diagram of f88 and f88M2e2-16 bacteriophages. (C, D) Characterization of CsCl-gradient-centrifuge purified f88M2e2-16 and f88ctr phages using coomassie blue staining and western blot. Two μg of f88M2e2-16 and f88ctr bacteriophages were loaded on SDS-PAGE and the corresponding Western blot was visualized using anti-pVIII mAb, anti-pIII mAb, anti-M2e mAb 37 and anti-M2e mAb 148, as indicated. MAb 37 is an anti-M2e IgG1 specifically recognizing amino acids 4–14 of human consensus M2e; mAb 148 is an anti-M2e IgG1 that specifically binds the conserved first nine amino acids of M2e. (E) Recombinant pVIII-M2e2-16 and f88 phages were separated using Tricine-SDS-PAGE with 15% acrylamide gel. Mouse anti-pVIII antibody was then used to probe protein samples in Western Blot. The upper faint protein band in the left lane corresponds to pVIII-M2e2-16 and is absent in f88 phages. The faster migrating and more intense band corresponds to wild type pVIII. (F) phage preparation titration using TG1 *Escherichia coli* strain containing F pilus. 100 μl bacteriophage containing 10^10^ tetracycline transducing units were diluted with 5x10^6^, 5x10^7^ and 5x10^8^ times and were plated on tetracycline-containing LB agar plates. Each colony forming unit represents one viable bacteriophage particle.

To construct viable filamentous phages displaying M2e, we genetically fused M2e2-16 (SLLTEVETPIRNEWG) to the N-terminus of the pVIII coat protein of bacteriophage f88. As starting material, we used f88 in which the information coding for VHEPHEFRHVALNPV is fused to the recombinant pVIII operon in the f88 phagemid. This influenza-irrelevant amino acid sequence containing the EFRH epitope of human β-amyloid peptide induced the production of anti-aggregating β-amyloid antibodies [39]. This construct, named f88ctr, was kindly provided by Dr. Beka Solomon (Tel-Aviv, Israel), and the donation was approved by Dr. G. Smith (University of Missouri). The coding information for M2e2-16 was introduced by conventional molecular biology techniques and the integrity of the M2e2-16-pVIII open reading frame in the resulting construct, named f88M2e2-16, was confirmed by Sanger sequencing (Fig 1A). F88ctr and f88M2e2-16 were transformed into competent *Escherichia coli* TG1 cells and the expression of recombinant pVIII was induced with 1 mM Isopropyl β-D-1-thiogalactopyranoside (IPTG) at 28°C for 4 hours. Bacteriophages were pelleted from the cleared supernatant by thorough mixing with PEG8000/NaCl solution to precipitate virions, which were subsequently collected by centrifugation at 18,600 g for 30 min at 4°C. The phage-containing pellet was resuspended in 30 ml Tris-buffered saline (TBS) pH 7.5. After clearing the resuspended phages by 10 min centrifugation at 18,600 g, the PEG8000/NaCl precipitation step was repeated. The final virion solution was further purified using CsCl gradient ultraspeed centrifugation in an SW41 rotor and an Optima L-90k ultracentrifuge (Beckman coulter, California, U.S.). For this, 4.83 g CsCl was added to 10.75 g of phage-containing TBS solution, transferred into a polyallomer tube and centrifuged at 209,490 g for 40 hours at 4°C. Bacteriophages were then collected by puncturing the tube with a 16-gauge needle just beneath the light-scattering band seen when shining a bright light downward into the tube. The bacteriophages were pelleted by centrifugation in a 60Ti rotor (Beckman coulter, California) at 251,000 g for 4 hours at 4°C and the resulting pellet was resuspended in 3.2 ml TBS.

Bacteriophages were titrated by determining the tetracycline-transducing units. For this, after gentle shaking for 5 min, TG1 *E*. *coli* bacteria were pelleted by centrifugation at 580 g for 10 min, resuspended in 20 ml 80 mM NaCl solution and gently shaken at 37°C for 45 min to starve the cells. Afterwards, ten-fold serial dilutions of filamentous phages were prepared in TBS and 20 μl bacteriophage suspension was added to 20 μl starved TG1 *E*. *coli* cells. After 10 min of incubation at room temperature, the mixture was diluted with 2 ml Luria broth (LB) medium containing 0.2 μg/ml tetracycline and incubated with gentle shaking for 40 min at 37°C. Cultures were then plated on LB agar plates with 20 μg/ml tetracycline and, following overnight incubation at 37°C, colonies were counted.

Two micrograms of purified f88ctr and f88M2e2-16 were analyzed by sodium dodecyl sulfate polyacrylamide gel electrophoresis (SDS-PAGE) followed by western blotting. Immunoblots were probed with anti-pIII mouse monoclonal antibody (mAb) (Progen, Sanbio B.V., the Netherlands), anti-pVIII mouse mAb (Progen, Sanbio B.V., the Netherlands) or with M2e-specific mouse mAb 37 (14C2-like) or 148 (specific for the N-terminal 9 amino acids of M2e). The tricine-SDS-PAGE method [40] was used for separation of recombinant pVIII from total pVIII subunits, and the band intensity was measured using ImageJ (National Institute of Health, U.S.).

### Propagation of influenza A viruses

Influenza A viruses were amplified on Madin-Darby canine kidney (MDCK) cells in serum-free cell culture medium (Dulbecco’s Modified Eagle medium (DMEM) supplemented with non-essential amino acids, 2 mM L-glutamine and 0.4 mM sodium pyruvate) in the presence of 2 μg/ml TPCK-treated trypsin (Sigma) at 37°C in 5% CO_2_. After 96 hour, the culture medium was collected, and cell debris was removed by centrifugation for 10 min at 2,500 g at 4°C, and the virus was pelleted from the supernatants by overnight centrifugation at 16,000 g at 4°C. The pellet was resuspended in cold sterile 20% glycerol in PBS, aliquoted and stored at −80°C.

### Immunization and Influenza A virus challenge

All mouse experiments were conducted according to the national (Belgian Law 14/08/1986 and 22/12/2003, Belgian Royal Decree 06/04/2010) and European (EU Directives 2010/63/EU, 86/609/EEG) animal regulations. Animal protocols were approved by the Institutional ethics committee of Ghent University (Eth. Com. No. 2012–032). All efforts were made to minimize the suffering of the animals. Specific-pathogen-free female BALB/c mice were obtained from Charles River (France) and immunized at the age of eight weeks. The animals were housed in a temperature-controlled room (biosafety level 2) with 14/10-h light/dark cycles and received food and water ad libitum. Mice were kept under specific pathogen-free conditions. The mice were immunized starting at the age of 78 weeks with 10^10^ f88M2e2-16 or f88ctr particles per mouse per immunization. The endotoxin levels were determined using ToxinSensor Chromogenic LAL Endotoxin Assay Kit (Genscript, U.S.) and were 0.033 EU/ml endotoxin for f88ctr and 0.024 EU/ml endotoxin for f88M2e2-16. As controls, we immunized mice with 5 μg of twotandem avian M2eHepatitis B core particles, 10 μg of three tandem human M2eHepatitis B core particles [41] or lipopolysaccharide (LPS)- free phosphate buffered saline (PBS). Antigens and PBS were formulated with incomplete Freund’s adjuvant (Millipore Corporation, U.S.) and injected intraperitoneally three times at three week intervals. Two weeks after the last immunization, all mice were intranasally challenged with mouse adapted influenza A virus. For mortality and morbidity monitoring we used 4 LD_50_ (lethal dose 50%) of A/Puerto Rico/8/34 (H1N1, abbreviated as PR8) (n = 10) or 4 LD_50_ A/Memphis/106/76 (H3N2) (n = 6). One LD_50_ of PR8 virus corresponds to 175 plaque forming units (pfu) and 1 LD_50_ of A/Memphis/106/76 virus to 1.2 × 10^5^ pfu. For lung virus titer assays, we used 1 LD_50_ PR8 (n = 3) or 1 LD_50_ A/Memphis/106/76 (n = 3). To mimic a natural infection, we used 0.2 LD_50_ A/Belgium/145/2009 pandemic H1N1 in 50 μl LPS-free PBS. One LD_50_ pandemic H1N1 virus corresponds to 9,500 pfu. Mice were weighed daily after lethal dose challenges, and animals that had lost 25% or more of their initial body weight (determined on the day of challenge) were euthanized by cervical dislocation.

### Histological analysis

Three mice per group from the groups immunized with f88M2e2-16, f88ctr or avian M2eHBc particles were euthanized 10 days after infection with 1 LD_50_ of PR8 virus. After intratracheal infusion of 1 ml OCT solution per lung (a formulation of water-soluble glycols and resins; Sakura, U.S.), the lungs were excised and immediately transferred into sealed containers, snap-frozen in liquid nitrogen and stored at -80°C until analysis. The lung samples were cut using a cryo-microtome (HM 560 Cryostat, Thermo Scientific, U.S.). Three sections from three different parts of the lungs were stained with hematoxylin and eosin (Sigma-Aldrich, U.S.) and examined microscopically unbiased by 2 board certified pathologists (SAY, AdB). The severity of the inflammation in the examined lung sections was scored on a scale of 0 to 5 (with 0.5 interval). Scores were given as absent (0), subtle (1), mild (2), moderate (3), severe (4), and massive (5).

### Serological analysis by enzyme-linked immunosorbent assay (ELISA)

Blood was collected from the lateral tail vein before vaccination, 10 days after each vaccination and 15 days after challenge. Titers of total M2e-specific serum IgG and f88- specific serum IgG were determined by ELISA using 96-well Maxisorp immuno-plates (Thermo, Nunc, U.S.) coated overnight with human consensus M2e2-24 peptide (Table 1) or purified f88ctr phages. All peptides were HPLC-purified and used at 2 μg/ml in carbonate buffer (3.39 g/L Na_2_CO_3_; 5.71 g/L NaHCO_3_; pH 9.6) at 50 μl/well at 37°C. After coating, plates were washed twice with PBS + 0.1% Tween20 and blocked with 1% bovine serum albumin (Sigma-Aldrich, U.S.) in PBS. The specific anti-M2e antibody titers in mouse serum were determined by incubating 1/3 serial dilutions in coated plates, starting with 1/100 dilution, for 1 hour. The wells were then washed and incubated with sheep anti-mouse IgG serum conjugated with horseradish peroxidase (HRP) (GE Healthcare UK Ltd.), or HRP-conjugated goat anti-mouse IgG1 and IgG2a (Southern Biotech, U.S.) for 45 min at room temperature. Finally, plates were washed and incubated with tetramethylbenzidine substrate (Sigma-Aldrich, U.S.) for 5 min and the reaction was stopped by adding 50 μl of 1 M H_2_SO_4_.

**Table 1:** List of different M2e sequences used in this study.

Present in	Codes	Sequence	Origin
M2e vaccine	f88-M2e2-16	SLLTEVETPIRNEWG	Human consensus
	VLP-1818	SLLTEVETPIRNEWGCRCNDSSD	Human consensus
	AvM2e-HBc	SLLTEVETPTRNEWECRCSDSSD	A/chicken/Vietnam/36/2004 (H5N1)
Influenza A virus	pdmM2e	SLLTEVETPTRSEWECRCSDSSD	A/California/7/2009 (H1N1)
	PR8M2e	SLLTEVETPIRNEWGCRCNGSSD	A/Puerto Rico/8/34 (H1N1)
	MemM2e	SLLTEVETLIRSEWGCRCNDSSD	A/Memphis/106/76 (H3N2)
	cNCM2e	SLLTEVETHTRNGWGCRCSDSSD	A/chicken/Nanchang/3-120/2001 (H3N2)
Peptide	M2e2-23	SLLTEVETPIRNEWGCRCNDSSD	Human consensus
	sOnM2e	SLLTEVETPTRNGWECRCSDSSD	A/swine/Ontario/42729A/01 (H3N3)
	cVNM2e	SLLTEVETPTRNEWECRCSDSSD	A/chicken/Vietnam/36/2004 (H5N1)
	sBelM2e	SLLTEVETPTRNGWECRYSGSSD	A/Swine/Belgium/1/1998 (H1N1)
	dVNM2e	SLLTEVETPTRNEWGCRCSDSSD	A/duck/Vietnam/NCVD-9/2007 (H5N1)
	cHKM2e	SLLTEVETLTRNGWGCRCSDSSD	A/Chicken/HongKong/258/1997 (H5N1)

### Hemagglutination inhibition (HAI) assay

Mouse sera were pretreated with four volumes of receptor-destroying enzyme (Sigma-Aldrich, U.S.) and incubated overnight at 37°C. Five volumes of filter-sterilized 1.5% sodium citrate solution was added and the mixture was heated at 56°C for 30 min. 1/10 volume of 50% chicken red blood cells was added to the sera followed by incubation for 1 hour at 4°C to pre-clear the sera. Samples were then centrifuged for 10 min at 1,000 g, and twofold serial dilutions of the supernatant were prepared and tested for HAI activity against A/Belgium/145/2009 (pandemic H1N1) as previously described [18].

### Determination of lung virus titers

Three mice from each immunization group were sacrificed at 6 days post 1 LD_50_ A/Memphis/106/76 infection and at 10 days post 1 LD_50_ PR8 infection. Lungs were excised and homogenized in 10% (w:v) PBS. Lung homogenates were cleared from debris by centrifugation for 10 min at 16,000 g. MDCK cells were seeded in triplicate at 2 × 10^4^ cells per well in a 96-well plate and infected with 50 μl of a 1/10-dilution series of the cleared lung homogenates. After 1 hour of incubation at 37°C, 100 μl serum-free medium (DMEM; 2 mM L-glutamine, Life technologies, Thermo Fisher Scientific, U.S.), supplemented with 10 μg/ml streptomycin, 10 U/ml penicillin (Hyclone, U.S.), 0.1 mM MEM non-essential amino acid (Gibco, Thermo Fisher Scientific, U.S.) and 2 μg/ml TPCK-treated trypsin. After seven days of incubation at 37°C, the presence of virus in the supernatant was assayed by measuring the hemagglutinating activity in the supernatant and using the method of Reed and Muench for calculation [42].

### Immunofluorescence microscopy of infected cells

MDCK cells, seeded at 5 × 10^4^ cells per well in a 96-well Black/Clear polystyrene high-content imaging plate with flat bottom (Cat. No. 353219, BD Falcon, U.S.), were mock infected or infected with PR8 (H1N1), A/Belgium/145/2009 (pandemic H1N1), A/Chicken/Nanchang/3-120/2001 (H3N2) or A/Memphis/106/76 (H3N2) at MOI 0.5 in serum-free medium. After 1 hour of incubation at 37°C, the cells were washed 3 times with LPS-free PBS and incubated with serum-free medium overnight at 37°C. The cells were then washed with LPS-free PBS and fixed with 1% paraformaldehyde (PFA) at room temperature for 30 min. Cells were blocked with 3% milk solution in PBS for 1 hour at room temperature and stained at room temperature for 2 hours with 1/300 diluted pooled pre-immune sera or with serum from mice immunized with avian M2eHBc, f88M2e2-16 or f88ctr. Then the cells were fixed again with 1% PFA at room temperature for 30 min. After permeabilization with PBS containing 0.15% glycine and 0.5% TritonX100 at room temperature for 15 min and again blocking with 3% milk solution for 1 hour at room temperature, goat anti-ribonucleoprotein polyclonal antiserum (BEI Resources, NIH, U.S.) was used to immuno-stain the cells. A mixture of Alexa Fluor 555 Donkey Anti-Mouse IgG and Alexa Fluor 488 Donkey Anti-Goat IgG (Life technologies, Thermo Fisher Scientific, U.S.) was used as fluorescently labeled secondary antibodies. The samples were visualized using a Leica TCS SP5 II confocal microscope (Leica Microsystems, Germany) with 63x magnification.

### Statistical analysis

To determine the statistical significance between values from two different groups, a two-tailed Student’s t-test was used. P value < 0.05 was considered to be statistically significant. For comparison of multiple groups, GraphPad Prism software was used for two-way ANOVA statistical analysis.

## Results

### Immunization with recombinant filamentous phages displaying truncated M2e induces M2e-specific serum IgG

We constructed recombinant filamentous bacteriophages that display M2e2-16 (SLLTEVETPIRNEWG) by genetically fusing them with the N-terminus of the major coat protein pVIII (Fig 1A and 1B). We used an f88 vector that expresses wild type pVIII as well as a recombinant version of pVIII [43–45]. Expression of the recombinant pVIII is controlled by an IPTG-inducible promoter, and in frame genetic fusion at the N-terminus of pVIII with heterologous sequences was accomplished by molecular cloning. We used the replicative form of a previously described recombinant f88 phage carrying a 15-mer peptide fused to the N-terminus of the recombinant pVIII operon in f88 as a control construct (ctr) and for introducing the M2e2-16 sequence [39] (Fig 1A). The resulting f88M2e2-16 construct was genetically stable and resulted in production of viable phages that multiplied to high titers in TG1 *E*. *coli* (Fig 1). pVIII is the major phage capsomer and makes up the helical structure of fd phages (Fig 1B). SDS-PAGE followed by Coomassie brilliant blue staining of purified f88ctr and f88M2e2-16 phages revealed one predominant band corresponding to pVIII protein originating from the wild type phage expression cassette (Fig 1C). The desired M2e2-16pVIII fusion protein was expected to migrate more slowly than wild type pVIII (predicted molecular weight 10,340 Da compared to 7,621 Da for wild type pVIII). Protein staining did not indicate the presence of an M2e2-16pVIII fusion protein, but western blot analysis using two different mouse monoclonal antibodies (mAb) specific for M2e, pVIII and pIII showed that the genetically inserted M2e2-16 sequence was present in purified f88M2e2-16 phage particles (Fig 1D). A more detailed western blot analysis of the recombinant M2e2-16pVIII phages was performed by separating purified phages in a 15% acrylamide gels using tricine-SDS-PAGE, followed by western blotting. Immunodetection with anti-pVIII mAb revealed the presence of a slightly slower migrating immunoreactive band in the f88M2e2-16 phages but not in f88 phages (Fig 1E). Based on the signal intensity of the immunoblot, we estimated that 510% of the incorporated pVIII proteins corresponded to M2e2-16 fusion proteins. We also generated an f88 construct with a variant M2e2-16 sequence (SLLTEVETPTRNEWE, corresponding to M2e of A/Vietnam/1203/2004 H5N1). However, this construct resulted in much lower phage titers than f88ctr and f88M2e2-16. Furthermore, the SLLTEVETPTRNEWE-pVIII fusion of those phages was poorly detectable by western blot, suggesting that the copy number of the recombinant pVIII fusion in the hybrid phages with the H5N1-derived M2e2-16 was much lower than that in the phages with human M2e2-16pVIII (data not shown). F88M2e2-16 and f88ctr were expressed and purified using the same procedures. The numbers of colonies in serially diluted phage suspension co-incubated with *E*. *coli* cells were similar on tetracyclinecontaining LB plates, indicating that the tetracycline transducing ability via F-pilus of *E*.*coli* is comparable for both recombinant f88 (Fig 1F).

**Fig 2:**
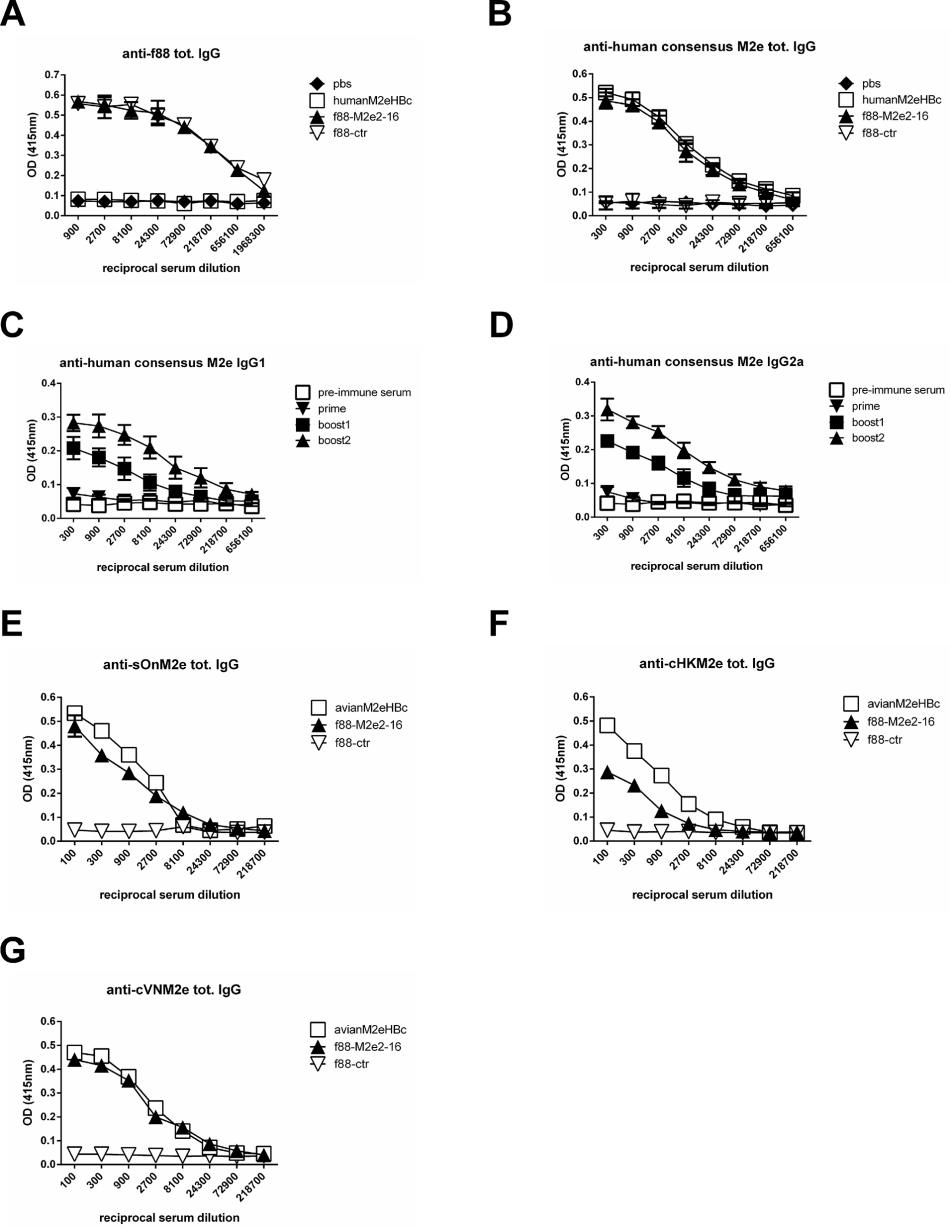
Immunization of mice with f88M2e2-16 phages results in robust anti-M2e specific serum IgG responses. (A-D) Groups of female BALB/c mice (n = 10 per group) were intraperitoneally vaccinated with antigens in the presence of incomplete Freund’s adjuvant via the intraperitoneal route. Purified phages were administered at 10^10^ tetracycline transducing units (f88M2e2-16 and f88ctr groups). As a positive control, one group was immunized with 10 μg M2eHBc per vaccination. A PBS plus adjuvant was included as a negative control. Blood was collected 10 days after priming and each boost injection and serum was prepared and tested in ELISA. (A) Anti-f88 total IgG titration following priming and boosting. (B) Anti-human consensus M2e total IgG following priming and boosting. (C) Anti-human consensus M2e IgG1 and (D) IgG2a endpoint titration following priming and boosting. Endpoint serum IgG titers after the third boost were determined from BALB/c mice that had been immunized with 10^10^ tetracycline transducing units of f88ctr, 10^10^ tetracycline transducing units f88M2e2-16 or 10 μg of avian M2e-HBc using ELISA plates coated with M2e peptides from different influenza A virus strains: (E) M2e from A/swine/Ontario/42729A/01 (sOnM2e), (F) M2e from A/chicken/HongKong/258/1997 (cHKM2e), (G) M2e from A/chicken/Vietnam/36/2004 (cVNM2e). In A-D error bars represent standard deviations.

We next assessed the immunogenicity of purified f88M2e2-16 phages in BALB/c mice. Mice were immunized with 10^10^ f88ctr or f88M2e2-16 phages per dose. We included mice immunized with M2eHBc as a positive control and mice injected with PBS as an additional negative control. All vaccines were adjuvanted with Incomplete Freund’s Adjuvant and were injected intraperitoneally into female BALB/c mice starting at the age of 68 weeks (n = 10 per group in the PR8-challenge experiment; n = 6 per group in the A/Memphis/106/76 (H3N2) challenge experiment). Vaccines were administered three times with three weeks interval. Ten days after each immunization, the mice were bled to test for IgG antibodies specific for M2e or f88 phage. Immunization with f88M2e2-16 induced robust serum anti-f88 IgG responses that were comparable in magnitude to those elicited by the f88ctr (Fig 2A). The humoral anti-M2e immune responses was determined by peptide ELISA and scored for total M2e-specific IgG, IgG1 and IgG2a. The second and third immunization with f88M2e2-16 boosted the M2e-specific serum IgG1 and IgG2a titers (Fig 2B–2D).

The first nine amino acid residues of M2 are fully conserved whereas the membrane proximal part of M2e is less conserved [33, 46]. We therefore tested the binding of the immune sera to different M2e peptides in ELISA. Monoclonal antibodies that are directed against the N-terminus of M2e recognize and potentially protect against all influenza A viruses whereas other antibodies that recognize more membrane proximal parts of M2e, such as mAb 14C2, recognize M2e from a subset of influenza A viruses [47, 48]. An overview of the M2e constructs and sequences used in this study is shown in Table 1. We compared the binding of immune sera to sBeM2e, cHKM2e and cVNM2e in peptide ELISA. Reactivity of f88M2e2-16 immune serum was comparable to that of immune sera from mice immunized with avian M2eHBc for M2e peptides sBeM2e and cVNM2e, and slightly lower for cHKM2e. The cHKM2e variant contains a proline at position 10 (Fig 2E–2G). The importance of proline 10 in M2 for target recognition and passive immune protection with M2e-specific mAbs has been documented by escape selection experiments in SCID mice [49]. Taken together, we conclude that immunization with hybrid recombinant filamentous phages displaying both M2e2-16pVIII as well as wild type pVIII capsomers, induces significant M2e-specific serum IgG levels.

### Immune serum raised by f88M2e2-16 immunization binds to cells infected with influenza A virus

**Fig 3:**
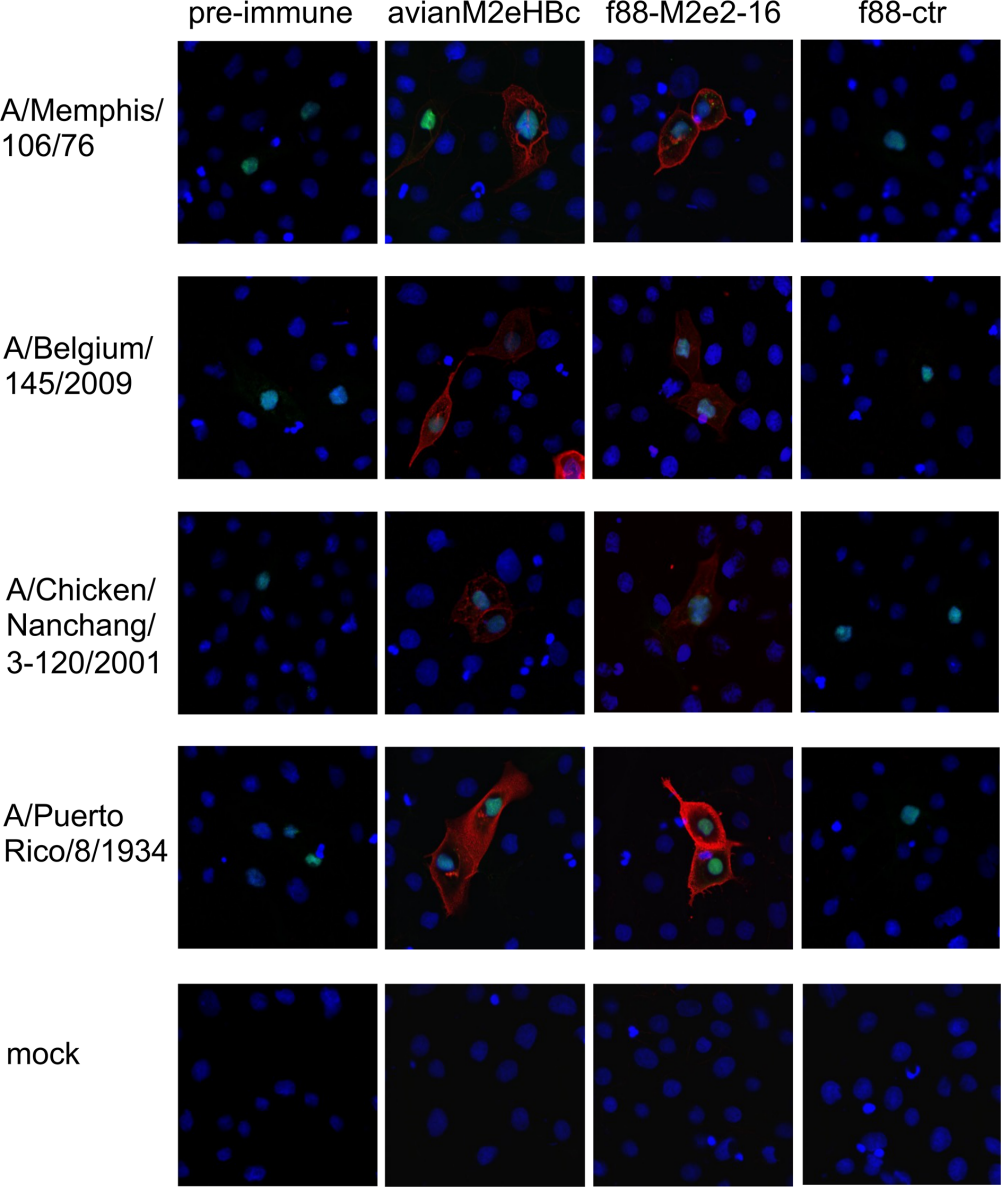
Immunization of mice with f88M2e2-16 phages induces anti-M2e specific serum IgG that binds to human and avian influenza A virus infected mammalian cells. Preimmune and immune sera collected after the third immunization with avian M2e-HBc, f88M2e2-16 or f88ctr were used to immuno-stain Madin-Darby canine kidney (MDCK) cells that had been infected with A/Memphis/106/76 (H3N2), A/Belgium/145/2009 (pandemic H1N1), A/chicken/Nanchang/3-120/2001 (H3N2), PR8 (H1N1) or mock infected. Alexa Fluor 555 Donkey anti-Mouse secondary antibody (red fluorescence) was used to reveal mouse IgG binding. An Alexa Fluor 488 labeled polyclonal goat anti-RNP staining (green fluorescence) was used to trace influenza A virus infected cells. All cells were stained with DAPI (blue fluorescence).

We next determined if immune sera raised by the recombinant M2e-displaying filamentous phages can recognize cells infected with influenza A virus, the likely targets of M2e-based humoral immunity [35]. MDCK cells were infected overnight with A/Memphis/106/76 (H3N2), A/Belgium/145/2009 (pandemic H1N1), A/Chicken/Nanchang/3-120/2001 (H3N2) or PR8 (H1N1) and then fixed and immune-stained with serum from mice that had been immunized three times with f88M2e2-16 or f88ctr phages. Pre-immune serum and serum from mice immunized with M2eHBc were included as negative and positive controls, respectively. As shown in Fig 3, serum from mice that had been immunized with f88M2e2-16 phages, specifically bound to infected cells, indicating that natural tetrameric M2 is specifically recognized by this serum.

### Protection by f88M2e2-16 vaccine against influenza A virus challenge

**Fig 4:**
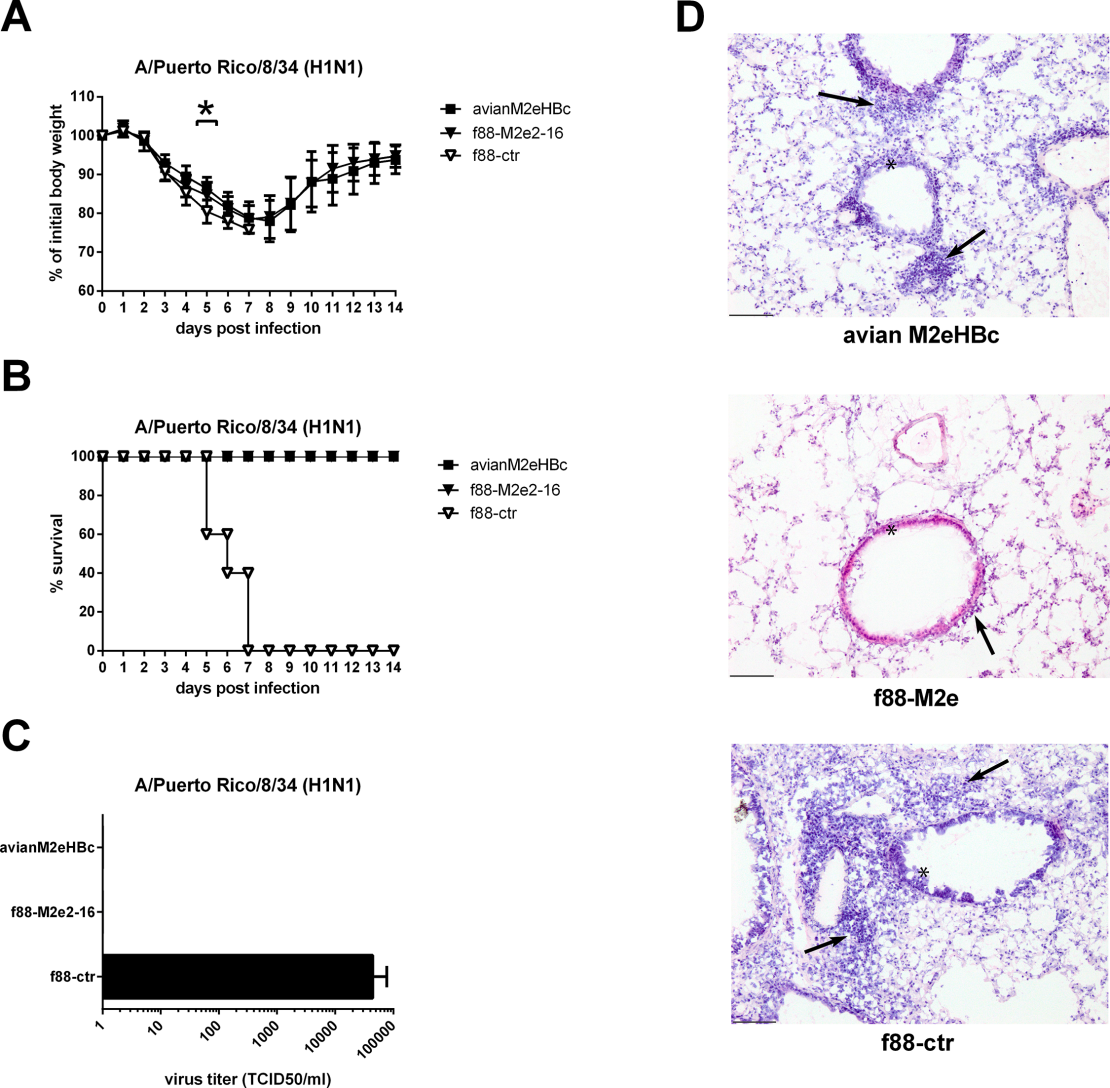
Vaccination with f88M2e2-16 protects against influenza A virus challenge. Six—eight weeks old female BALB/c mice (n = 10 per group) were immunized with 10^10^ f88M2e2-16 bacteriophage particles, avian M2eHBc or f88ctr via the intraperitoneal route for 3 times at 3 weeks interval in the presence of incomplete Freund’s adjuvant. Two weeks after the last boost, groups of mice were challenged with 4 LD_50_ mouse adapted PR8 H1N1. (A) Morbidity and (B) mortality following challenge. (C) Lung virus titers in lung homogenates isolated on day 10 after challenge of mice vaccinated as in (A) with 1 LD_50_ of mouse adapted PR8 virus. (D) Mice vaccinated as in A were sacrificed on day 10 after challenge with 1 LD_50_ of mouse adapted PR8. Ten μm-thick lung section were prepared and stained with Hematoxylin—eosin. Representative slides magnified 10 times are shown. Black arrows indicate bronchial infiltration of immune cells.

To evaluate the cross-protection efficacy of immunization with f88M2e2-16 phages, vaccinated mice were challenged two weeks after the last boost with 4 LD_50_ of mouse-adapted PR8 (H1N1) (Fig 4). Morbidity and mortality were monitored daily after challenge to evaluate protection. All mice that had received f88M2e2-16 or M2eHBc vaccination experienced transient body weight loss but survived the challenge (Fig 4A and 4B). In contrast, all mice immunized with f88ctr or PBS died or had to be euthanized after H1N1 (Fig 4A and 4B). We also determined lung virus titers ten days after PR8 infection. For this, three mice in each group were challenged with 1 LD_50_ of PR8. Mice immunized with f88M2e2-16 or M2eHBc had cleared the virus from their lungs, whereas mice immunized with f88ctr still had residual virus (Fig 4C). Finally, we performed histopathology analysis on cryosections of lung samples from mice that had been challenged with PR8 virus. Hematoxylin and eosin staining showed much more immune cell infiltration in the lungs of the f88ctr group than in the f88M2e2-16 and avian M2eHBc groups (Fig 4D). The inflammation scores (see Materials and Methods for scoring) for the respective lung sections were 1.16 (f88M2e2-16 group), 2.0 (M2eHBc group) and 2.50 (F88-ctr group).

**Fig 5:**
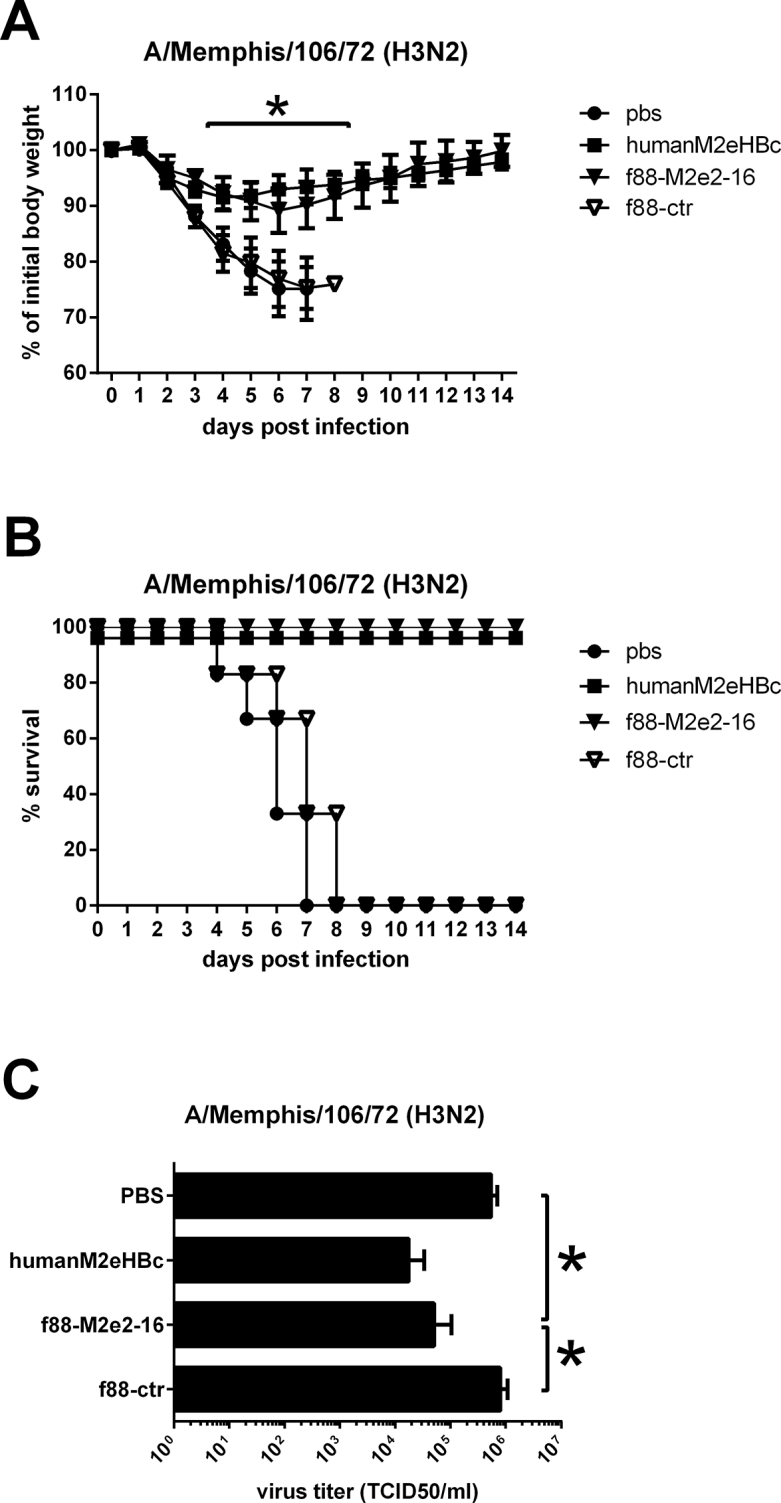
Vaccination with f88M2e2-16 protects against challenge with an influenza A virus that carries a Pro10 to Leu change in M2e. Six—eight weeks old female BALB/c mice (n = 6 per group) were immunized with 10^10^ f88M2e2-16 bacteriophage particles, 10^10^ f88ctr bacteriophage particles, 10 μg human M2eHBc or PBS. Immunization was done via the intraperitoneal route, in the presence of incomplete Freund’s adjuvant and 3 times with 3 weeks interval. Two weeks after the last boost, mice were challenged with 4 LD_50_ of mouse adapted A/Memphis/106/76 H3N2. (A) Morbidity and (B) mortality following challenge. (C) Lung virus titers determined on day 6 after challenge with 1 LD_50_ of mouse adapted A/Memphis/106/76 H3N2 virus.

We repeated the protection experiment using A/Memphis/106/76 (H3N2) as a challenge virus. Body weight loss was significantly different between the f88M2e2-16 and M2eHBc vaccinated mice on the one hand and the PBS and f88ctr vaccinated mice on the other hand (Fig 5A, p< 0.05, two-way ANOVA). F88ctr immunized mice did not survive challenge with this H3N2 virus, whereas M2eHBc and f88M2e2-16 immunized animals all survived (Fig 5B). We also determined lung virus titers on day 6 after challenge with 1 LD_50_ of A/Memphis/106/76 virus and found significantly lower titers in mice immunized with f88M2e2-16 or M2eHBc than in f88ctr and PBS immunized animals (Fig 5C).

### M2e-specific serum IgG and HAI responses following challenge

**Fig 6:**
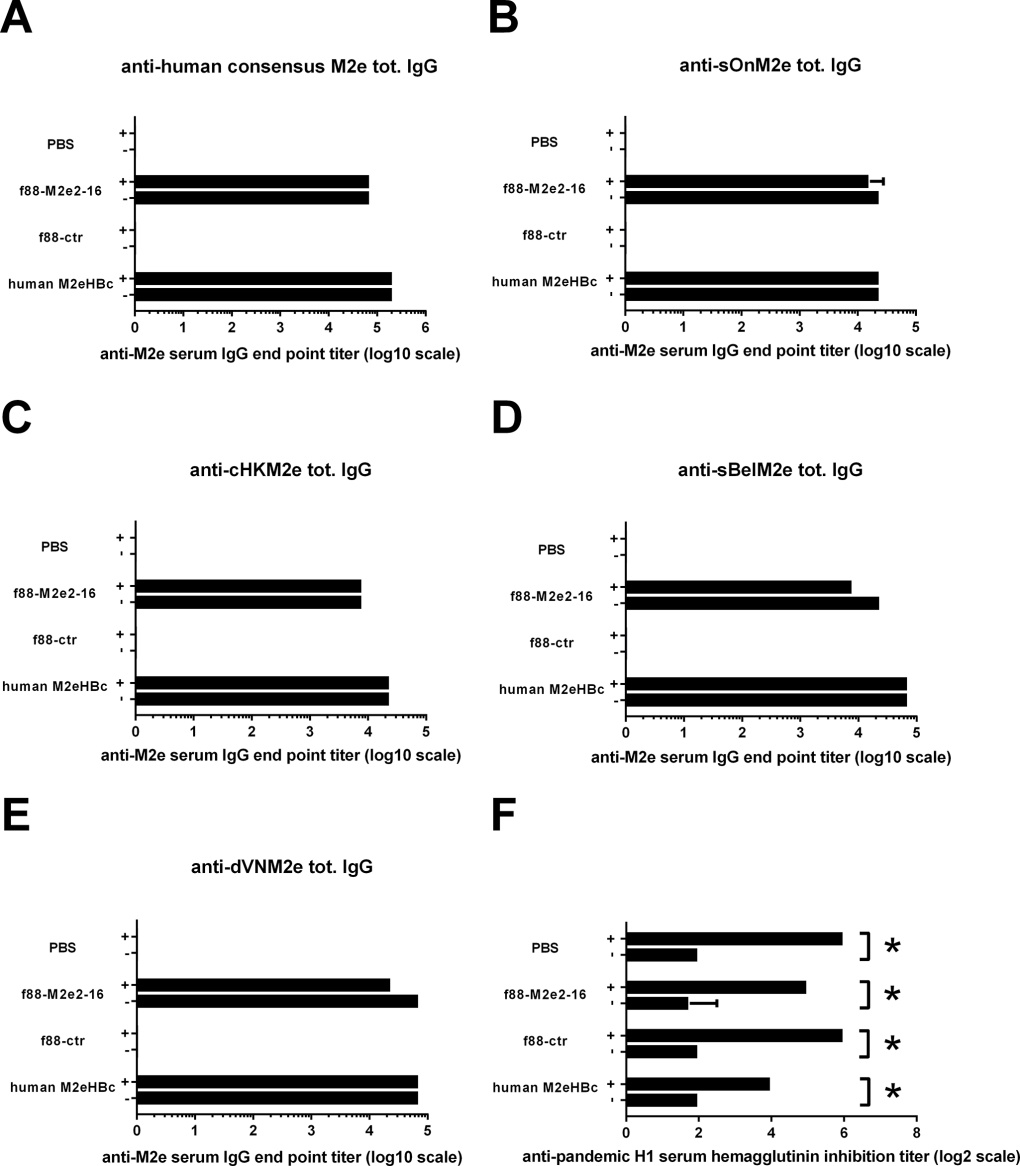
Challenge of f88M2e2-16 immunized mice does not boost the anti-M2e serum IgG response. BALB/c mice (n = 10) were vaccinated with f88M2e2-16, f88-ctr, human M2eHBc or PBS three times in the presence of incomplete Freund’s adjuvant. Three weeks after the last boost, mice were challenged with 0.2 LD_50_ of mouse-adapted pandemic H1N1 2009 virus. Serum was collected 10 day after the last booster immunization (-) and 15 days after infection (+). The anti-M2e serum IgG response was determined by ELISA coated with (A) human consensus M2e (M2e2-23), M2e from (B) A/swine/Ontario/42729A/01 (sOnM2e), (C) M2e from A/chicken/Hong Kong/258/1997 (cHKM2e), (D) M2e from A/swine/Belgium/1/1998 (sBelM2e) and (E) M2e from A/duck/Vietnam/NCVD-9/2007 (dVNM2e). (F) Hemagglutination inhibition titers against pandemic H1N1 2009 virus before and after infection of mice. Error bars represent standard deviations.

It was recently reported that natural infection of humans with A/Belgium/145/2009 (H1N1) pandemic virus was associated with a recall M2e-directed serum response in approximately half of the monitored individuals [19]. We investigated whether a sublethal infection with mouse-adapted H1N1 2009 pandemic virus of control, f88M2e2-16 and M2eHBc immunized mice, would alter M2e-specific IgG levels in serum. Serum samples were collected 15 days after the last immunization and 15 days after challenge with 0.2 LD_50_ of H1N1 2009 pandemic virus and tested by peptide ELISA. The plates were coated with different M2e peptides (Table 1), including human consensus M2e, cHKM2e, sOnM2e, sBelM2e and dVNM2e. Infection of PBS or f88ctr immunized mice did not induce detectable M2e-specific serum IgG titers, regardless of the peptide used for coating (Fig 6). Infection of mice immunized with f88M2e2-16 or M2eHBc did not significantly change the anti-M2e IgG titers, regardless of the M2e type peptide used for coating (Fig 6). The anti-M2e IgG titers in post-infection antiserum from all groups were similar or lower than the pre-infection antiserum, so the IgG titers 15 days after infection were not enhanced by a viral infection. Meanwhile, all mice had HAI titers after infection (Fig 6F). We conclude that immunization with f88M2e2-16 induces protective M2e-specific serum IgG that are not boosted by challenge with H1N1 2009 pandemic virus infection.

## Discussion

M2e of influenza A viruses is conserved and is considered as an antigen for the development of a universal influenza vaccine. To enhance the immunogenicity of M2e peptides, it is usually chemically or genetically coupled to a large enough carrier [20–23, 50]. Here, we analyzed the capacity of bacteriophage f88 to display M2e. F88 phages are filamentous and composed of 2700 copies of pVIII that form a helical structure around the single-stranded circular genomic DNA [51]. Importantly, f88 phages do not kill their host but behave as a persistent infection that slows down bacterial growth, with new phages continuously budding from the infected *E*. *coli* cells. We constructed a new M2e-based influenza A vaccine by genetic fusion of amino acids 2–16 of M2e to a fraction of the major coat protein pVIII capsomers. The resulting f88M2e2-16 phages are viable, and grow to high titers in F^+^ *E*. *coli* cells. In addition, immunization of mice with purified f88M2e2-16 phages elicited M2-specific IgG antibodies and protected these mice against challenge with influenza A viruses of different subtypes.

Incorporation of heterologous sequences as fusion partners with pVIII into the budding phages, depends in part on the size and nature of the heterologous peptide. There is a correlation between the copy number of recombinant pVIII in hybrid virions and the phage assembly rate. The influence of the foreign insert on the processing rate by the leader peptidase that cleaves off the secretion signal from pro-pVIII, is an important determinant of the incorporation efficiency of a heterologous pVIII fusion as a capsomer into the budding phage particles [52]. Endemann and Model proposed that insertion of more than six to eight amino acids at the N-terminus of pVIII would make the interaction between pVIII and pVII impossible and would lead to failure of the viral assembly [53]. We constructed M2e-displaying filamentous phages using the f88 vector, which contains a recombinant pVIII operon controlled by a Tac promoter and wild type pVIII. Our attempts to incorporate full-length M2e as a pVIII fusion into the filamentous phage were unsuccessful. Therefore we decided to use a truncated version of M2e (M2e2-16). By using tricine-SDS-PAGE and western blotting with anti-pVIII monoclonal antibody, we estimated that the display percentage of M2e2-16-pVIII was approximately 5–10% (Fig 1E).

Immune sera from f88-M2e2-16 immunized mice reacted with many M2e variant peptides in ELISA and with the cell surface of mammalian cells that had been infected with influenza A viruses expressing different M2e variants (Figs 2 and 3). We suspect that this broad reactivity is attributable to the induction of antibodies that are primarily directly against the N-terminal part of M2e. This part, SLLTEVET, is nearly absolutely conserved in influenza A viruses.

The M2e2-16 peptide includes EVETPIRN and monoclonal antibodies specific for this sequence have been shown to protect mice against PR8 virus challenge [54]. However, it was previously reported that the M2e2-16 peptide adjuvanted with complete/incomplete Freund’s adjuvants did not elicit antibodies in mice against full-length M2e [55]. These authors showed that a physically linked HA-derived T helper epitope was required to induce M2e-specific antibodies following immunization with the truncated M2e2-16 peptide. Since immunization with f88M2e2-16 phages resulted in robust M2e-specific serum IgG responses, it is likely that in our model, T helper epitopes are provided by the structural proteins in the phage particles. It is also possible that anti-pVIII antibody responses contributed to the immunogenicity of M2e during booster immunizations with f88-M2e2-16. With approximately 95% of pVIII still being wild type in f88-M2e2-16 phages, the antibody response to pVIII was comparable to that in f88-ctr immunized mice (Fig 2A). For each immunization with 10^10^ f88-M2e2-16 particles, only approximately 4 ng of M2e2-16 was injected. In comparison, we used 5 μg of M2e-HBc particles as a positive control, which corresponds to approximately 1,2 μg of M2e antigen. Despite this 300 fold difference in amount of M2e present in either vaccine, M2e-specific serum IgG responses were very comparable in these groups (Fig 2B, 2E and 2G). The pVIII-specific antibodies may well have contributed to enhanced activation of B cells by forming immune complexes with f88-M2e2-16 phages, which could then be efficiently recognized and processed by antigen presenting cells. In addition, we speculate that the single stranded DNA genome of the f88 phages have powerful adjuvant activity by stimulating *e*.*g*. Toll like receptor 9.

We administered the f88M2e2-16 phage combination in the peritoneum and using a strong adjuvants. This route and adjuvant are not suitable for human use. Therefore, additional routes and adjuvants (e.g. MF59) should be explored before considering clinical application of this experimental M2e-vaccine. An important advantage of phage based vaccines is there economical production cost, which could facilitate their use for veterinary use.

The infection-permissive M2e-based vaccines allow limited virus replication which leads to the induction of T cell responses directed against the conserved internal proteins of influenza A virus. In contrast, in experimental animal models, conventional influenza vaccines hamper the development of such cross-reactive T cell immunity [18, 56]. We found that intraperitoneal immunization with f88M2e2-16 phages reduced the immune cell infiltration on day 10 post PR8 infection (Fig 4D) and conferred full protection against challenge with a lethal dose of PR8 (H1N1) or A/Memphis/106/76 (H3N2) virus. Our histological analysis of the unprotected f88ctr mice concords with an early report showing that on day 10 after infection with PR8 virus there is massive lung infiltration of immune cells [57]. Moreover, the polyclonal anti-M2e antibodies in immune serum were capable of binding to M2e peptides derived from human, swine or avian influenza viruses. M2 is sparsely present on influenza virions but much more abundant on the surface of infected cells. We demonstrated that immune sera from mice immunized with f88M2e2-16 phages bound MDCK cells infected with influenza A virus, indicating that IgG in the immune sera binds to both human and avian-like tetrameric M2 on infected cells. This implies that anti-M2e antibodies in f88M2e2-16 immune sera could help to clear cells infected with influenza A virus by antibody-dependent cell-mediated cytotoxicity (ADCC) or phagocytosis (ADPC) [35].

The protection by M2e based vaccines are mediated mainly by M2e specific IgG antibodies and partly by also local M2e specific secreted IgA [20, 32, 58–60]. The efficacy of M2e vaccines in M2e specific humoral immunity induction determines the protection levels against influenza A virus infection. With an aggressive immunization protocol used in this *in vivo* mice experiment, the f88M2e2-16 phage induced dramatically enhanced protective antibody levels after the second and the third shot and conferred full protection against lethal dose challenge, which means that this vaccine has potential to be applied for further human clinical trial. Furthermore, previously there were no evidence of toxic effects on the tested animals [43, 61, 62].

We conclude that filamentous phages expressing M2e can be used to induce M2e-specific serum IgG antibodies that protect against a lethal dose of virus in the mouse model. This approach is economical and very easy to scale up.
